# Supplementary figures and images for: Inferring gene regulatory network from single-cell transcriptomes with graph autoencoder model
Source: PLoS Genet. 2023 Sep 13;19(9):e1010942. doi: 10.1371/journal.pgen.1010942 (PMC10519590; doi:10.1371/journal.pgen.1010942)

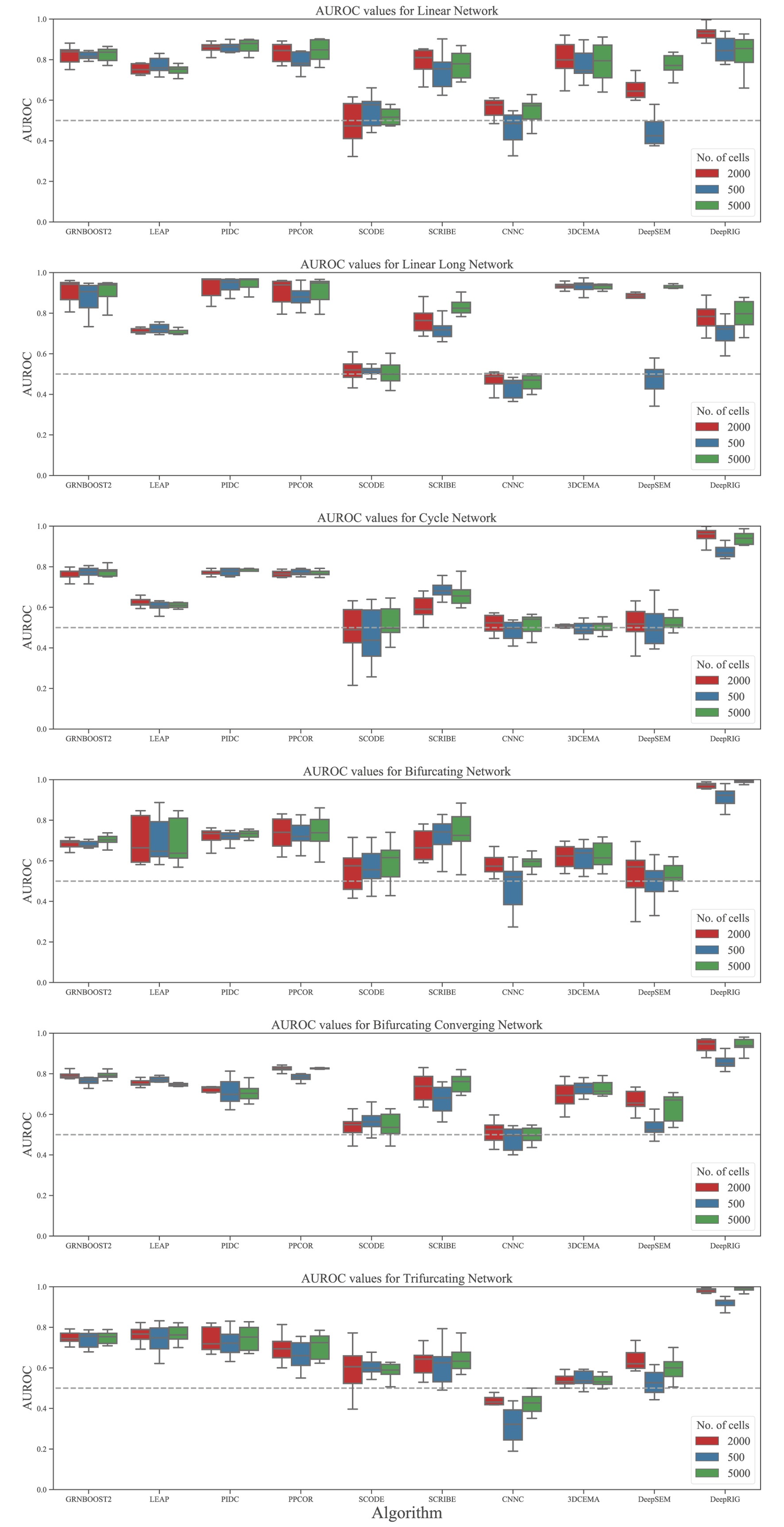

Supplement: S1 Fig — Each subfigure corresponds to one of the six synthetic networks. Each box of one method represents the values of 10 repeat runs. Red, blue and green respectively denote to the in silico datasets with 500, 2000, and 5000 cells. The gray dotted line represents the performance of the random predictor. (TIF) [file pgen.1010942.s001.tif]

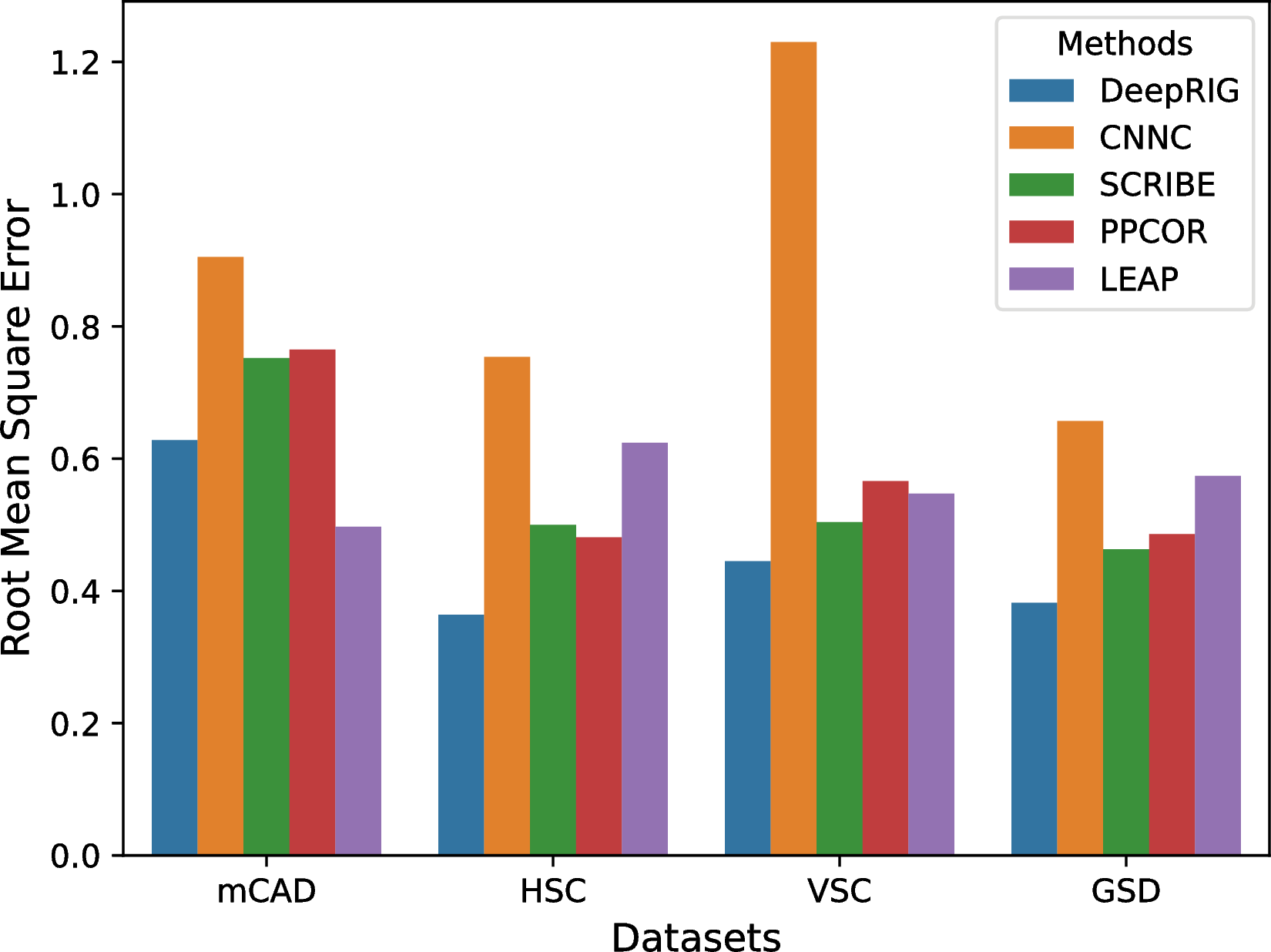

Supplement: S2 Fig — The value of the Y-axis represents the Root Mean Square Error (RMSE) between the predicted signed GRNs and the ground truth. The bar charts for each color represent the performance of a method. (TIF) [file pgen.1010942.s002.tif]

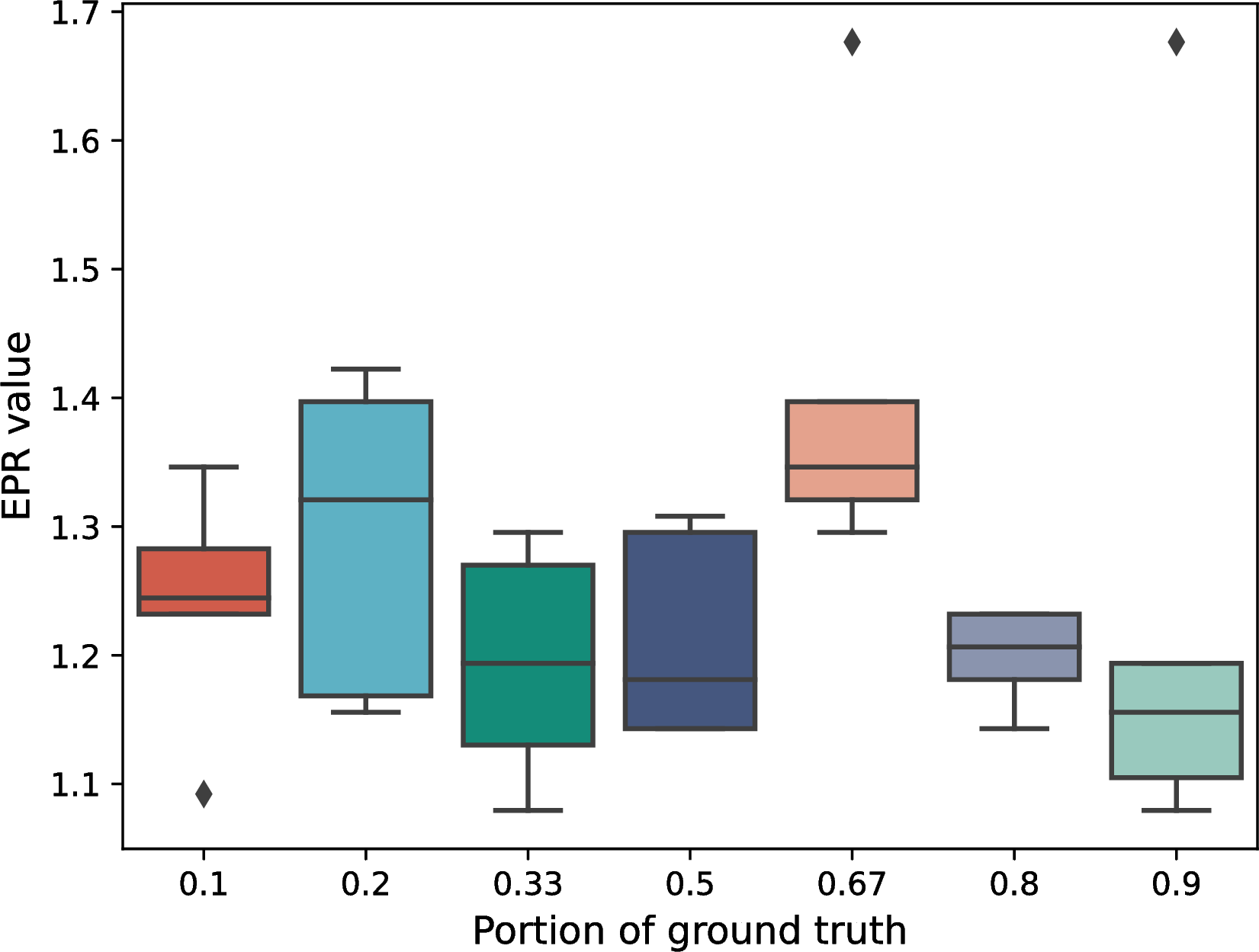

Supplement: S3 Fig — The portion of all the ground truth for training is 0.1, 0.2, 0.33, 0.5, 0.67, 0.8 and 0.9 in the seven columns as indicated by the X-axis labels. We performed five repeated experiments on the mESC dataset for each portion, with different random seeds used in each repetition. (TIF) [file pgen.1010942.s003.tif]

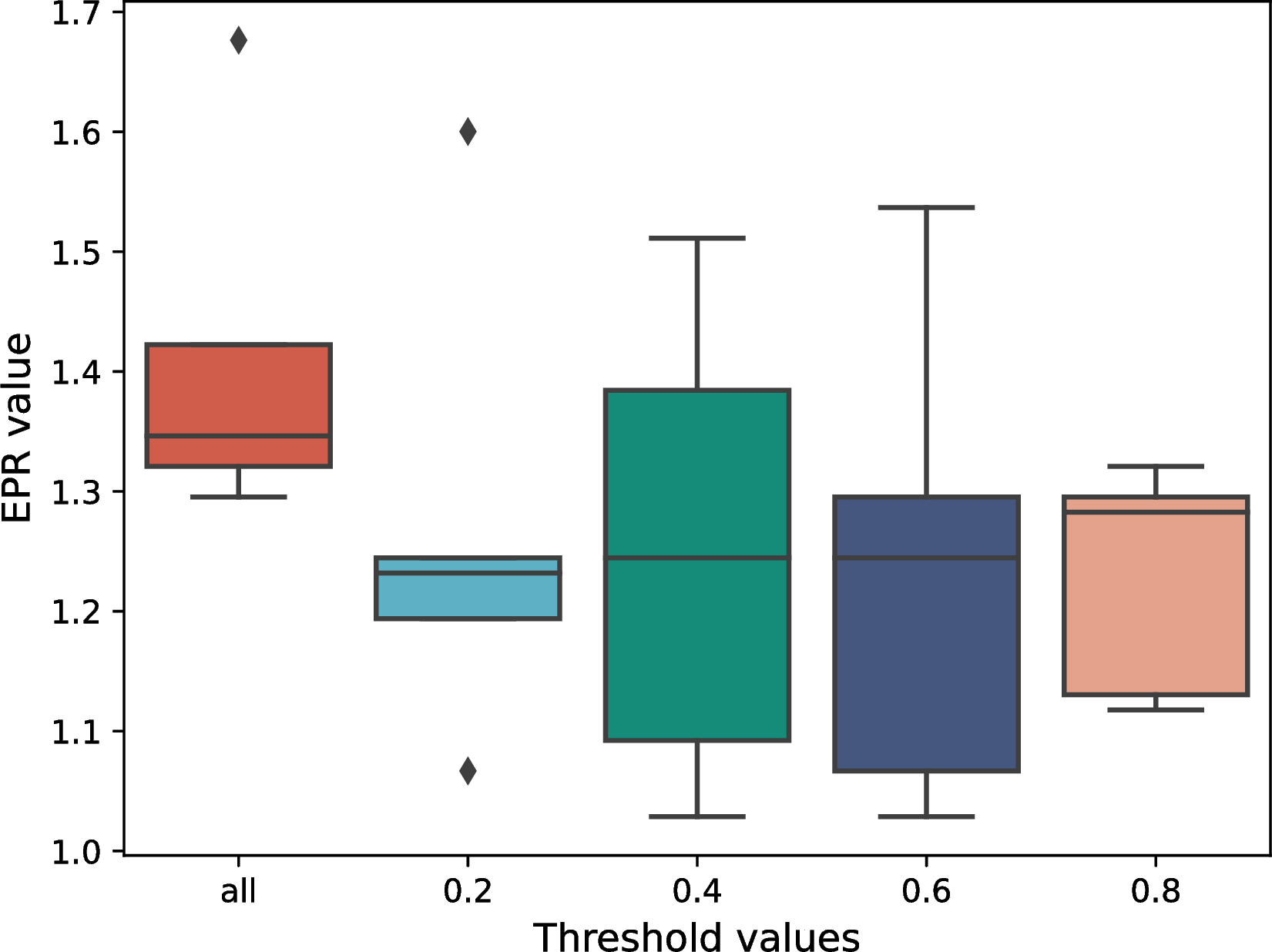

Supplement: S4 Fig — The threshold values for building WGCN are 0, 0.2, 0.4, 0.6, and 0.8 in the five columns as indicated by the X-axis labels. We performed five repeated experiments on the mESC dataset for each threshold value, with different random seeds used in each repetition. (TIF) [file pgen.1010942.s004.tif]

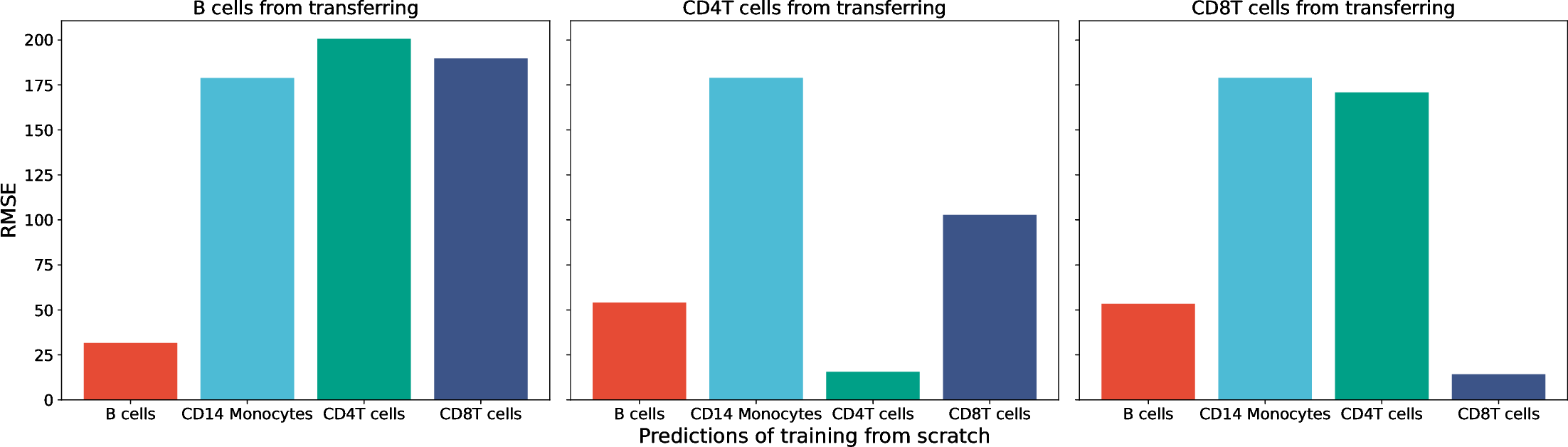

Supplement: S5 Fig — The value of the Y-axis represents the Root Mean Square Error (RMSE) between the predicted results from transferred models and the inferred results from trained models from scratch. The bar charts for each color represent a cell type. (TIF) [file pgen.1010942.s005.tif]

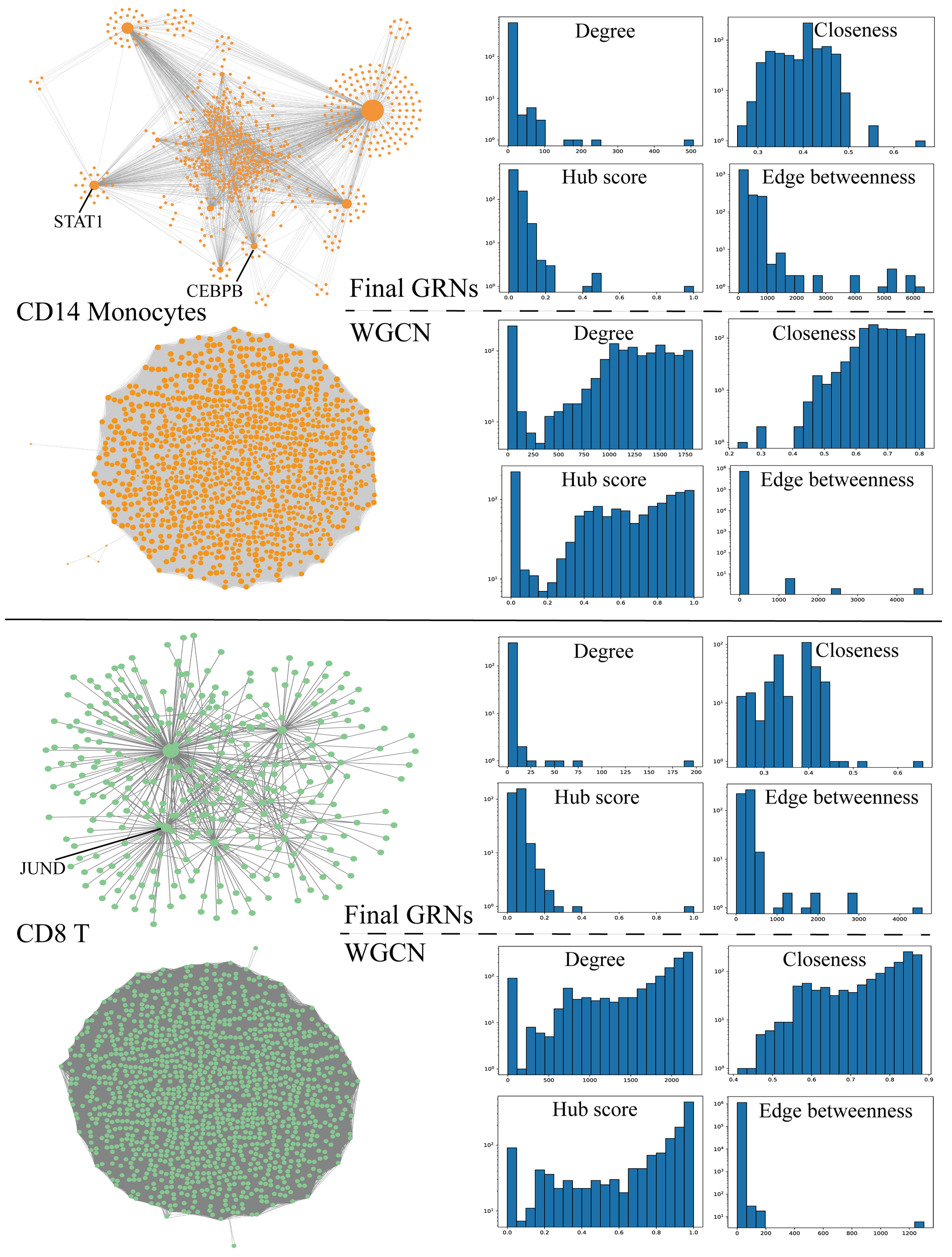

Supplement: S6 Fig — Two cell types, CD14monocytes and CD8T cells, are investigated respectively. In each subgraph, top and bottom represent the final predicted GRNs and WGCN, respectively. Left and right represent the visualization and the distribution of several topological properties of the network, respectively. (TIF) [file pgen.1010942.s006.tif]

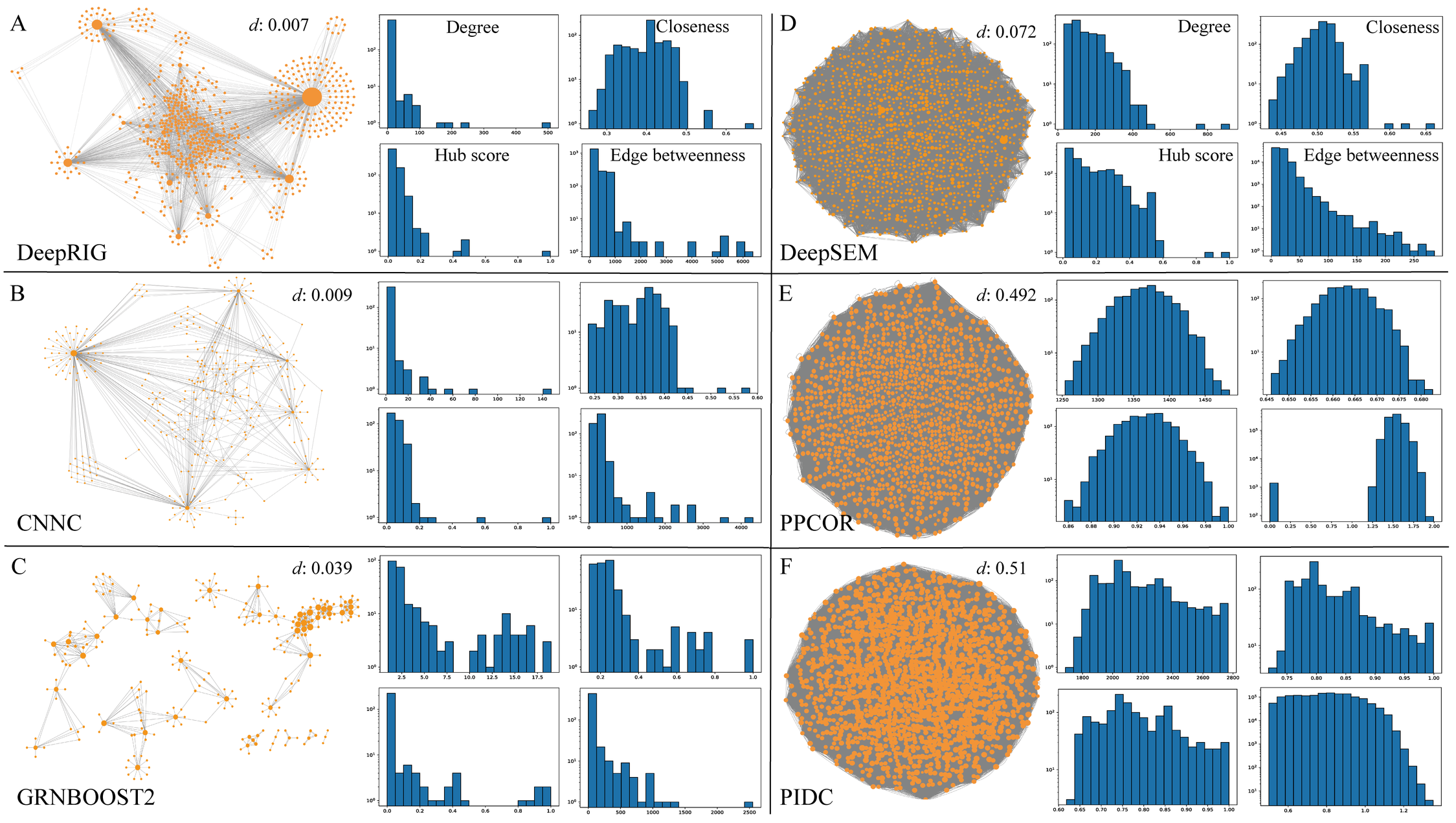

Supplement: S7 Fig — The visualization (left) and the distribution of several topological properties (right) of the inferred GRNs by (A) DeepRIG, (B) CNNC, (C) GRNBOOST2, (D) DeepSEM, (E) PPCOR and (F) PIDC, respectively, revealed differences. (TIF) [file pgen.1010942.s007.tif]

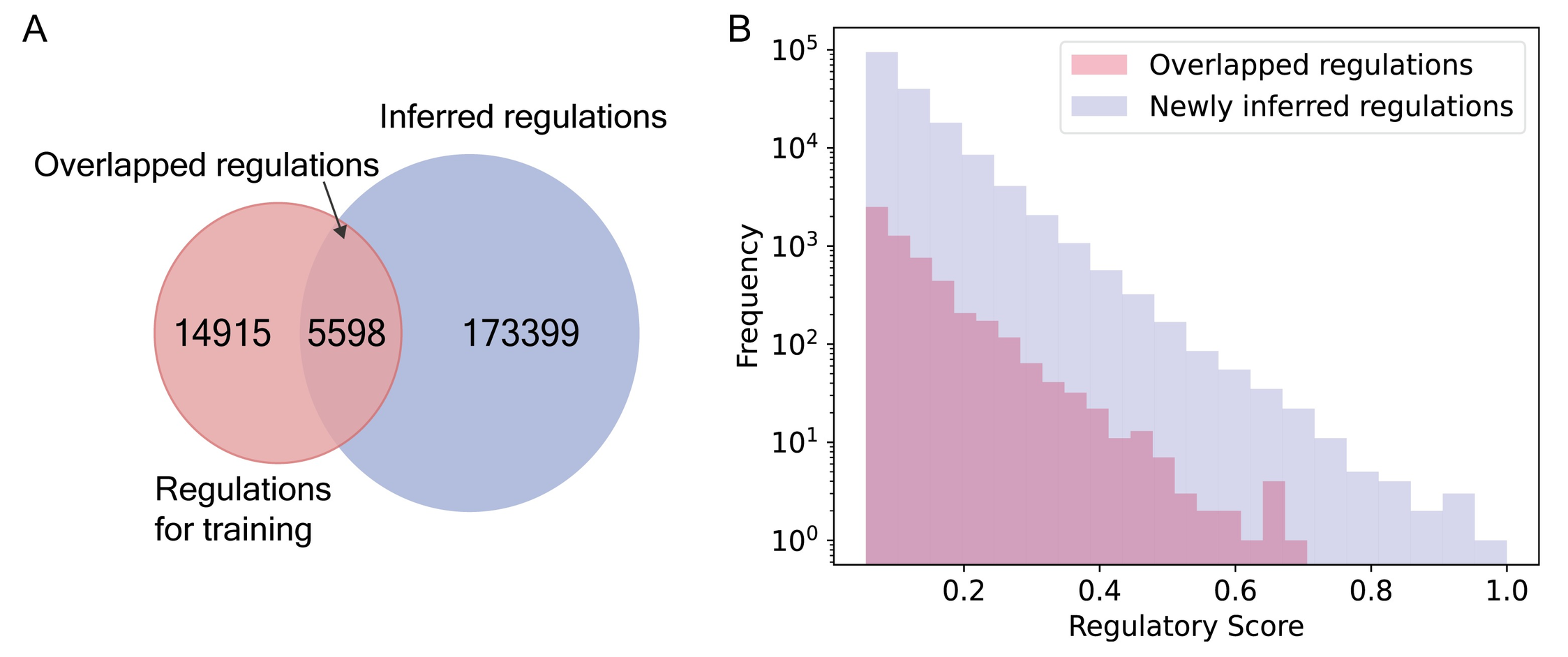

Supplement: S8 Fig — (A) The overlap between the inferred regulations and the ground truth regulations used for training. (B) The distributions of the regulatory scores from newly inferred regulations and the overlapped regulations. (TIF) [file pgen.1010942.s008.tif]

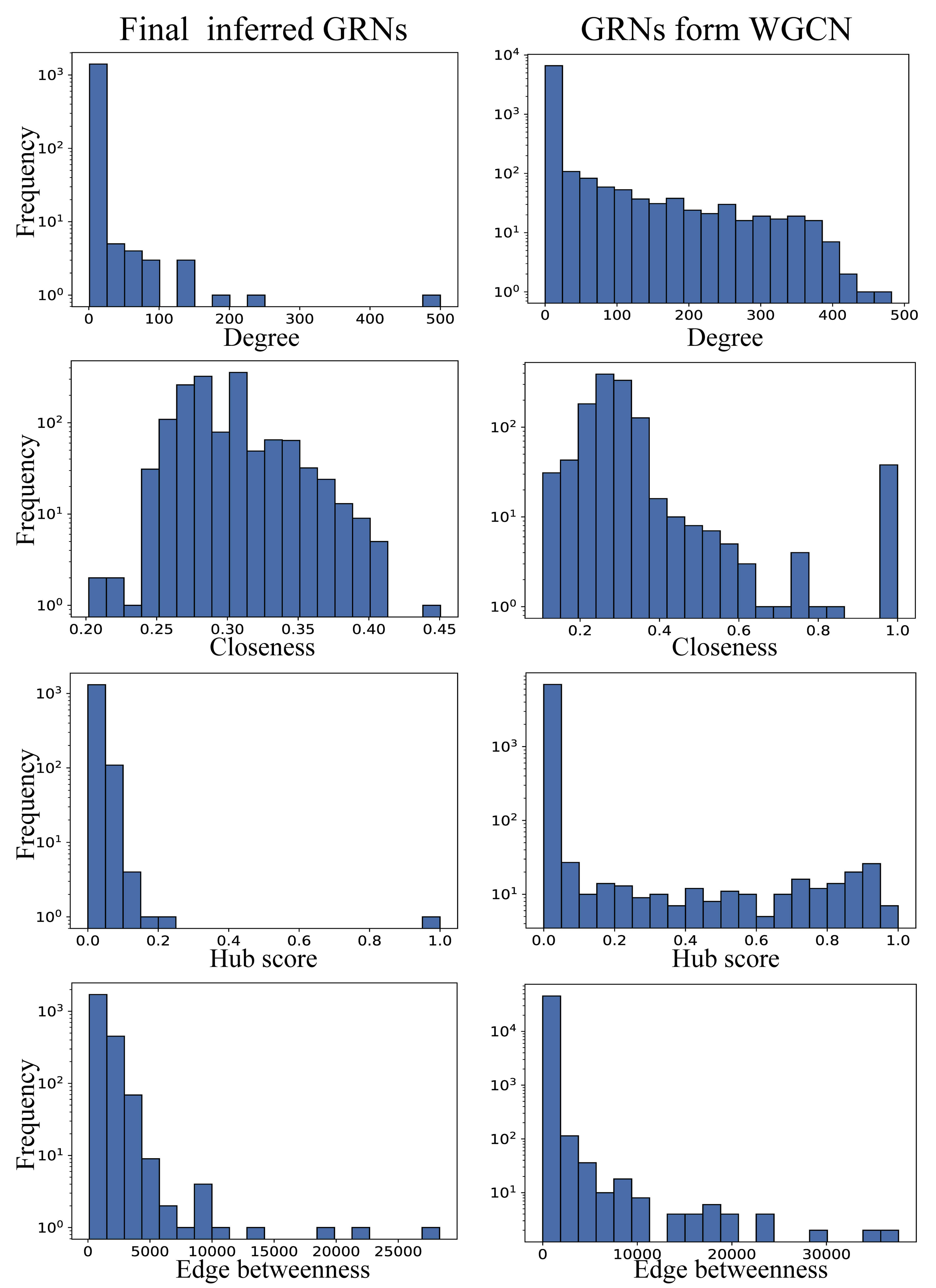

Supplement: S9 Fig — Left and right columns represent the distribution of four topological properties of the final inferred GRNs and the network from WGCN, respectively. (TIF) [file pgen.1010942.s009.tif]
