# Supplementary material for: Inferring gene regulatory network from single-cell transcriptomes with graph autoencoder model
Source: PLoS Genet. 2023 Sep 13;19(9):e1010942. doi: 10.1371/journal.pgen.1010942 (PMC10519590; doi:10.1371/journal.pgen.1010942)
Supplement: Attachment — Submitted filename: Response2reviewer.pdf [file pgen.1010942.s019.pdf]

We would like to express our sincere appreciation to the editors and the reviewers for their valuable time and thoughtful comments on our manuscript. Their constructive feedback and insightful suggestions have greatly contributed to the enhancement of our manuscript. We have carefully considered each comment and made extensive modifications to ensure that our paper meets the requirements and addresses all concerns raised. Below, we provide point-by-point responses to the reviewers' comments.

The reviewers' comments are shown in **black** and our responses are in **blue**.

Reviewer #1:

The manuscript entitled "Inferring gene regulatory network from single-cell transcriptomes with graph autoencoder model" introduces a graph-based deep learning model called DeepRIG, which is employed on single-cell RNA-sequencing data to infer cell type-specific GRNs. This approach offers the advantage of capturing local and global regulatory structures underlying gene expression, addressing a limitation often overlooked by other GRN inference methods. The performance of DeepRIG is extensively assessed against prior practices for GRNs inference on both in silico and real-cell regulatory networks, showing the benefit of the graph-based deep learning method. In particular, DeepRIG presents accurate cell type-specific GRNs inference for PBMCs and the identification of novel regulators for breast cancer.

I have a few questions:

1. When constructing a WGCN by measuring Spearman's correlation coefficients of each gene pair, the authors defined the edge weight with the absolute value of correlation coefficients. Why did the author adopt this definition strategy rather than other strategies, such as different thresholds? That's what I'm very interested in.

Authors' response: We thank the reviewer for raising this important point. The selection of a threshold is crucial in WGCNA. However, there is no consensus on the optimal threshold for all tasks, as it depends on the specific situation and analysis objective. In this study, we chose to retain all the correlation coefficients instead of adopting other predefined strategies. This approach was motivated by the expectation that it would capture a broader range of potential regulatory information than other strategies. We further compared the model performance under different thresholds of WGCN to verify our expectations (below and in Supplementary Fig 4). The model has the highest median EPR value when preserving all the edges of WGCN compared to other soft thresholds. This suggests that a WGCN that retains all weights does provide more potential regulatory information in our model.

Manuscript changes:

-Addition of Supplementary Fig 4:

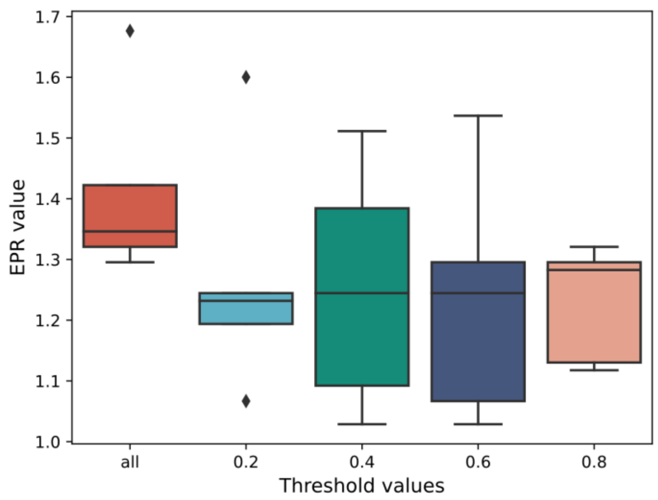

-Addition of the following text: "The selection of a threshold is crucial in WGCNA. However, there is no consensus on the optimal threshold for all tasks, as it depends on the specific situation and analysis objective. In this study, we chose to retain all the correlation coefficients instead of adopting other predefined strategies. This approach was motivated by the expectation that it would capture a broader range of potential regulatory information than other strategies (Fig S4)."

2. While the authors have summarized the six real-cell scRNA-seq datasets, their details and the corresponding ground truth of TF-target interactions still need to be well described, such as the number of TF and target genes in each dataset and the number of links in the corresponding ground truth.

Authors' response: We thank the reviewer for raising this important point. We have added the detailed clarifications for each real-cell scRNA-seq dataset, including the number of TFs and target genes, the number of links, average degrees per gene and TF, in the supplementary Table 1.

Manuscript changes:

-Addition of Supplementary Table 1:

Datasets	# of cells	# of Target genes	# of TFs	# of Links	# avg degree per gene	# avg degree per TF
hESC	758	876	34	4526	5.17	133.12
mESC	383	950	77	21315	22.44	276.82
mDC	456	801	20	756	0.94	37.8
mHSC-E	1072	691	29	11557	16.73	398.52
mHSC-L	848	544	16	4398	8.08	274.88
mHSC-GM	890	618	22	7364	11.92	334.73

* Note that "#" denotes the number.

3. It needs to be clarified how much of the results in the PBMC case study are new predictions from DeepRIG and how much is already present in the hTFtarget interactions used for training.

Authors' response: We thank the reviewer for this question. We have clarified the number of newly predicted regulations and the number of pre-existing regulations for training in the predicted regulations. And it can be seen in Supplementary Table 6.

Manuscript changes:

-Addition of Supplementary Table 6:

Datasets	# of predicted	# of newly predicted	# of pre-existing	Density of pre-existing
CD14Monocyte	638	441	197	0.309
BC	183	131	52	0.284
CD4T	34	25	9	0.265
CD8T	483	339	144	0.298

4. The authors have introduced several advantages of their proposed approach. Yet, to facilitate its usage, the authors may also discuss its limitations and potential caveats.

Authors' response: We thank the reviewer for raising this important point. To provide a balanced discussion of our method, we added the following commentary to the main text regarding limitations and potential caveats.

Manuscript changes:

Addition of the following text: "However, our method presents several limitations. Although DeepRIG is easily applied and adaptable as long as ChIP-seq data is available, it is unable to guide the model training in the absence of specific ground truth data. Additionally, using only scRNA-seq data as a basis for inferring GRNs can provide information on intracellular gene expression levels, it may not be sufficient for understanding the specific mechanisms of gene regulation. Therefore, the introduction of multi-omics single-cell data, especially scATAC-seq data, can provide more comprehensive and detailed information to help more accurately infer the topology and mechanisms of GRNs. The two limitations mentioned above will be the focus of our future work."

5. The authors need to mention any previous work on identifying regulons from scRNA-seq data, while it is also a practical approach to infer GRNs indirectly.

Authors' response: We thank the reviewer for raising this point. We briefly introduced and discussed several previous works on identifying regulons from scRNA-seq data in the main text.

Manuscript changes:

Addition of the following text: "Furthermore, methods including SCENIC, IRIS3, and CeRIS have been developed to identify the active regulons of specific cell types and to further reveal underlying GRNs in diverse diseases."

6. Some abbreviations in the manuscript do not have their complete spelling when they first appear, such as "scRNA-seq data" in Line 16 in the section of Abstract.

Authors' response: We thank the reviewer for raising this point. We checked through the whole manuscript and added the complete spelling of some abbreviations when they first appear.

Reviewer #2:

Jiacheng Wang et al. employed a graph autoencoder-based method to infer gene regulatory networks from single-cell RNA sequencing data. The model aims to elucidate the global regulatory structure rather than merely the regulatory relationships between gene pairs. I believe this study is one step further on GRN reconstruction and for genomic research. However, the following concerns need to be resolved to underscore the value of this study:

1. In the introductory section, since the authors have already mentioned tools that utilize RNA velocity and ODE models to infer gene regulatory networks, it would also be relevant to discuss very briefly the following works that employ similar data/models that could to some extent infer GRNs:

Qiu, X., et al. (2022). "Mapping transcriptomic vector fields of single cells." Cell.

Schiebinger, G., et al. (2019). "Optimal-Transport Analysis of Single-Cell Gene Expression Identifies Developmental Trajectories in Reprogramming." Cell.

Authors' response: We thank the reviewer for raising this point. We briefly introduced and discussed several previous works that utilize RNA velocity and ODE models to analyze the regulatory dynamics and to some extent infer GRNs.

Manuscript changes:

Addition of the following text: "Qiu et al. integrates RNA dynamics modeling, robust vector field reconstruction, and geometric analyses to construct and interpret single-cell transcriptomic vector fields, enabling insights into cell-fate transitions and genetic perturbations." And "Schiebinger introduced Waddington-OT, an innovative approach that uses developmental time courses from single-cell RNA sequencing to infer ancestor-descendant fates, model regulatory programs, and gain insights into diverse developmental processes, including reprogramming."

2. The impact of the presented research appears constrained due to the reliance on pre-existing "gold-standard" networks for training data. Although I understand that the model only needs to be fed with a fraction of known interactions, I think it would enhance the value of this tool if the authors could demonstrate that a trained model can be transferred to other cell types, conditions, or even datasets. The transferability of the method is somewhat demonstrated using the breast cancer dataset, where the model is trained with GRN of normal breast cells. This point is, however, not highlighted or further explored enough in my opinion. For instance, it would be compelling to see if the model trained on one or multiple cell types of the PBMC dataset could be applied to a different, untrained cell type.

Authors' response: We appreciate the valuable suggestion from the reviewer. To address the constraints of relying on a part of the gold standard networks, we conducted further investigations to assess the transferability of our model across different cell types. Following the reviewer's insightful recommendation, we trained our model on CD14 Monocytes and subsequently transferred it to three other cell types. And we found that our model presents good transferability across different cell types when comparing the predicted results from transferring to those obtained from each cell type from scratch.

For instance, in the case of B cells, we calculated the Root Mean Square Error (RMSE) between the predicted results obtained from transferring and those derived from training B cells from scratch (Below and Supplementary Fig. 5). Notably, the RMSE between the two models within B cells was significantly

lower compared to the RMSE between the transferred results from B cells and the results of CD14 monocytes trained from scratch, as well as the results of the other two cell types trained from scratch. This analysis suggests that the transferred results from B cells align more closely with the predicted results obtained from training B cells from scratch, rather than being heavily influenced by the training on CD14 monocytes.

Furthermore, we have provided a comprehensive list of the identified regulators from both the transferred model and the model trained from scratch (below and Supplementary Table 8). In the top-5 regulators predicted from the transferring, three regulators were also predicted from scratch, among which PAX5 and EGR1 have already been validated as cell-type-specific regulators for B cells (see Supplementary Table 7). Additionally, one of the regulators identified through the transferring process, EBF1, has been previously reported as an essential TF for maintaining B cell identity and preventing alternative fates in committed cells [1].

Manuscript changes:

-Addition of Supplementary Fig. 5 (below):

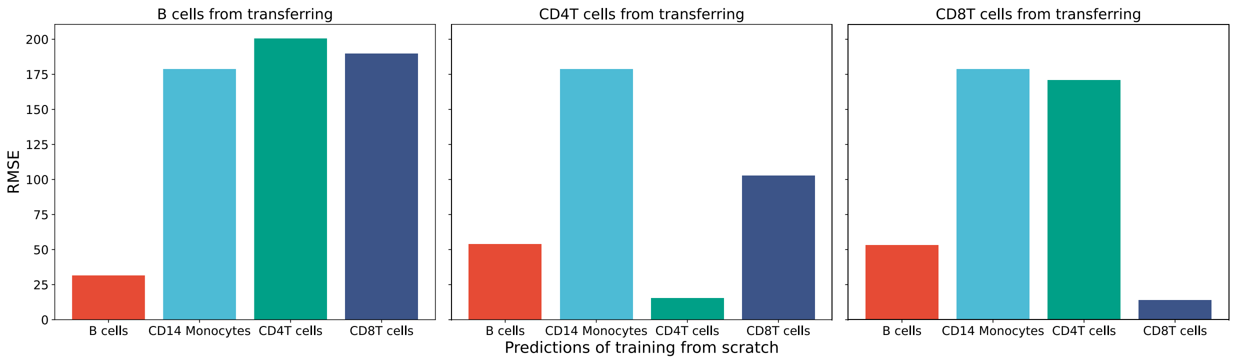

-Addition of Supplementary Table 8 (below):

Cell type	Identified regulators from trained models from scratch	Identified regulators from transferred models
B cells	EGR1, EGR2, TBX21, MEF2C, GATA2, BACH2, STAT1, MYC, NFE2, PAX5, RUNX3, FCER1A	PAX5, EGR1, MAX, KIAA0125, BACH2, P2RX5, SPIB, EBF1
CD4T cells	GPR82, BATF, TRBC1, MYB, SPI1, EGR1, LINC01550, SCGB3A1	EGR2, MYB, MAX, EGR1, JUND, RUNX3, BATF, CEBPB
CD8T cells	BACH2, NFE2, SPI1, MAX, GATA2, MEF2C, GATA3, ATF3	MYC, STAT1, JUND, MAX, RUNX3, TRABD2A, SPIB, RP11-127B20.3, ATF3, MEF2C, NFE2, GATA3

-Addition of the following text: “We conducted further investigations to assess the transferability of our model across different cell types. We trained our model on CD14 Monocytes and subsequently transferred it to three other cell types. And we found that our model presents good transferability across different cell types when comparing the predicted results from transferring to those obtained from each cell type from scratch.

For instance, in the case of B cells, we calculated the Root Mean Square Error (RMSE) between the predicted results obtained from transferring and those derived from training B cells from scratch (Fig. S5). Notably, the RMSE between the two models within B cells was significantly lower compared to the RMSE between the transferred results from B cells and the results of CD14 monocytes trained from scratch, as well as the results of the other two cell types trained from scratch. This analysis suggests that the transferred results from B cells align more closely with the predicted results obtained from training B cells from scratch, rather than being heavily influenced by the training on CD14 monocytes. Furthermore, we have provided a comprehensive list of the identified regulators from both the transferred model and the model trained from scratch (Table S8)."

3. In the TNBC dataset it seems to me that 14,915 interactions are used to train the model, while it predicted 173,399 interactions, which is 10+ times higher. Are the trained interactions all included in the predicted interactions? How confident are the newly predicted interactions? Is there any example of predicted interactions that are not in the training data validated through other experiments?

Authors' response: We thank the reviewer for raising this important point. We generated a Venn diagram (Supplementary Fig 8A) to visualize the overlap between the inferred GRNs and the interactions used for training the model. About 37.5% of the ground truth interactions (5598 interactions) used for model training were present in the final inferred GRNs. DeepRIG ranked the inferred regulations based on their regulatory scores. The interactions with higher scores are considered more reliable and confident. Furthermore, we displayed the distribution of their regulatory scores using a histogram, providing an overview of the confidence levels of the predicted interactions (Supplementary Fig S8B). We found that most of the newly inferred regulations are assigned the same or higher predicted scores compared to the overlapped interactions, indicating greater confidence in the newly predicted associations. Among the newly predicted interactions, an interesting finding was the regulation of the HIF1α pathway by XBP1. This prediction was validated by Chen et al.[2] through a series of experiments, including invasion assays, subcutaneous xenograft experiments, motif analysis on in-house ChIP-seq data, survival analysis, RT PCR analysis and immunoblot analysis. Furthermore, DeepRIG inferred that ZNF217 regulates the expression of KLF4, FTO, and ALKBH5, and is, in turn, regulated by HIF1α. Remarkably, Zhang et al.[3] performed RT-qPCR analysis, immunoblot assay and immunohistochemical analysis, and demonstrated that ZNF217 co-interacts with ALKBH5, functioning as a mediator to control the expression of KLF4 and NANOG involved in the post-transcriptional mechanism of HIF1α.

Manuscript changes:

-Addition of Supplementary Fig 8 (below):

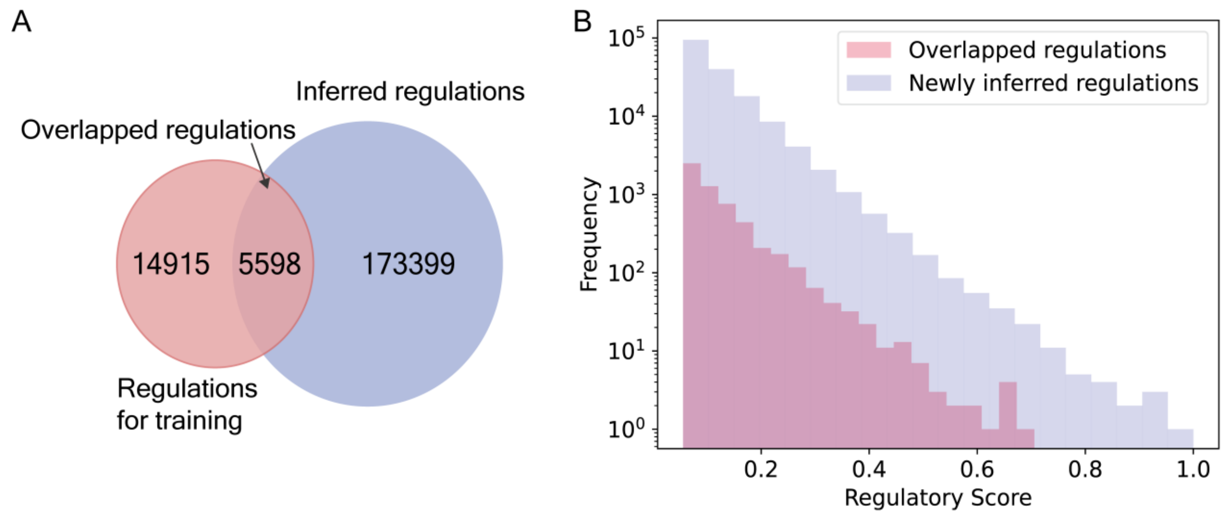

-Addition of the following text: "We then investigated the overlap between the inferred GRNs and the interactions used for training the model (Fig S8). About 37.5% of the ground truth interactions (5598 interactions) used for model training were present in the final inferred GRNs (Fig S8A). Furthermore, we found that most of the newly inferred regulations are assigned the same or higher predicted scores compared to the overlapped interactions, indicating greater confidence in the newly predicted associations (Fig S8B). Among the newly predicted interactions, an interesting finding was the regulation of the HIF1α pathway by XBP1. This prediction was validated by Chen et al.[2] through a series of experiments, including invasion assays, subcutaneous xenograft experiments, motif analysis on in-house ChIP-seq data, survival analysis, RT PCR analysis and immunoblot analysis. Furthermore, DeepRIG inferred that ZNF217 regulates the expression of KLF4, FTO, and ALKBH5, and is, in turn, regulated by HIF1α. Remarkably, Zhang et al.[3] performed RT-qPCR analysis, immunoblot assay and immunohistochemical analysis, and demonstrated that ZNF217 co-interacts with ALKBH5, functioning as a mediator to control the expression of KLF4 and NANOG involved in the post-transcriptional mechanism of HIF1α. The findings emphasize the critical role of these altered regulatory interactions in the development and progression of breast cancer."

4. When using simulated and real data for the performance comparison, my understanding is that the underlying networks and Chip-seq validated interactions are used to train deepRIG. This makes the comparison not completely fair since other methods may not be trained on network structures but only on expression data (e.g. deepSEM). This should be addressed or clearly explained in the result section. In addition, the authors may consider using the simulated data to demonstrate what portion of all the interactions should be used to train the model in order for it to make accurate predictions.

Authors' response: We thank the reviewer for raising this important point. We acknowledge that comparing supervised and unsupervised methods directly may raise concerns about fairness, as they have different access to prior knowledge. It is indeed true that supervised learning methods, such as DeepRIG, CNNC, and 3DCEMA, benefit from the utilization of ground truth ChIP-seq data, which can significantly enhance their performance in GRN inference. We humbly recognize the advantage that prior knowledge provides to supervised methods in terms of performance improvement.

At the same time, it is important to highlight that this advantage does not diminish the value of unsupervised methods. Unsupervised approaches, despite lacking the benefit of ChIP-seq data, still play a crucial role in inferring GRNs and have their own merits in handling diverse biological scenarios where prior knowledge may be limited or unavailable. By presenting the comparison in a balanced and objective manner, we aim to underscore the complementary strengths of both supervised and unsupervised methods in GRN inference.

We have clearly stated and explained it in the result section.

Manuscript changes:

-Addition of following text: “We acknowledge that comparing supervised and unsupervised methods directly may raise concerns about fairness, as they have different access to prior knowledge. It is indeed true that supervised learning methods, such as DeepRIG, CNNC, and 3DCEMA, benefit from the utilization of ground truth ChIP-seq data, which can significantly enhance their performance in GRN inference. We humbly recognize the advantage that prior knowledge provides to supervised methods in terms of performance improvement. At the same time, it is important to highlight that this advantage does not diminish the value of unsupervised methods. Unsupervised approaches, despite lacking the benefit of ChIP-seq data, still play a crucial role in inferring GRNs and have their own merits in handling diverse biological scenarios where prior knowledge may be limited or unavailable.”

In addition, the authors may consider using the simulated data to demonstrate what portion of all the interactions should be used to train the model in order for it to make accurate predictions.

Authors’ response: We appreciate the valuable suggestion from the reviewer. To ensure what portion of all the interactions should be used to train the model for making more accurate predictions, we further investigated on mESC scRNA-seq dataset. We used different portions of all the ground truth interactions to train our model and utilized the rest to test the model (below and Supplementary Fig 3). We found that DeepRIG reached highest EPR median value when using 67% ground truth interactions for training, compared to other portions. Notably, the reason we used mESC scRNA-seq data instead of simulated data is that the number of ground truth interactions from mESC is largest among all benchmark datasets, which can ensure there is enough much interactions to test the model no matter what portion of ground truth is used for training.

Manuscript changes:

-Addition of Supplementary Fig 3:

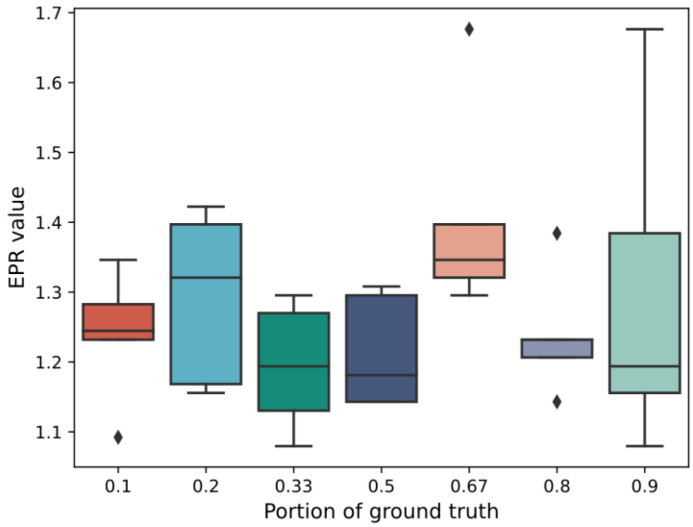

-Addition of following text: "To ensure what portion of all the interactions should be used to train the model for making more accurate predictions, we further investigated on mESC scRNA-seq dataset. We used different portions of all the ground truth interactions to train our model and utilized the rest to test the model (Fig S3). We found that DeepRIG reached highest EPR median value when using 67% ground truth interactions for training, compared to other portions."

5. In figure 8B and related paragraphs, the authors should briefly explain the meanings of betweenness and pagerank scores and what biological properties do they describe about the genes.

Authors' response: We thank the reviewer for raising this point. Bwtweenness and pagerank scores are two important network analytics metrics that assess the importance or influence of nodes in a network. Specifically, betweenness centrality measures the intermediacy of a node in the network, while PageRank measures the global importance of a node based on the network's link structure and importance propagation. Genes with high betweenness and PageRank scores are more likely to be considered as important hub genes or regulators. We have added above descriptions into figure 8 and related paragraphs.

Manuscript changes:

-Addition of following text: "Bwtweenness and pagerank scores are two important network analytics metrics that assess the importance or influence of nodes in a network. Specifically, betweenness centrality measures the intermediacy of a node in the network, while PageRank measures the global importance of a node based on the network's link structure and importance propagation. Genes with high betweenness and PageRank scores are more likely to be considered as important hub genes or regulators."

Reviewer #3:

The study introduces DeepRIG, a graph-based deep learning model for inferring gene regulatory networks (GRNs) from single-cell RNA sequencing data. Unlike traditional methods that only consider pairwise gene regulatory relationships, DeepRIG captures the global regulatory structure by transforming gene expression data into a co-expression mode to build a prior regulatory graph. It then employs a graph autoencoder model to embed the global regulatory information into gene latent embeddings and reconstruct the GRN. Benchmarking results show that DeepRIG outperforms existing methods in accurately reconstructing GRNs on completely synthetic and simulated regulatory networks. The model was applied to human peripheral blood mononuclear cells and triple-negative breast cancer samples.

While the manuscript introduces a novel method to improve GRN inference, it lacks clarity on how the WGCNA network and the final GRN differ (and whether those differences are biologically meaningful at all). There are also concerns in the way the comparison to other methods is presented, as discussed below.

Major comments:

1. There is no information on how the WGCNA network and the final GRN differ, especially in the real data presented in figures 7 and 8, where most of the networks have a single central node. The authors should presented clear data on how the GRN differs between the "prior regulatory graph" defined by the WGCNA and the final inferred GRN. Please present what nodes and edges change in figure 7 and 8 if one were to reconstruct the network just using the weights of WGCNA.

Authors' response: We appreciate the valuable suggestion from the reviewer. We explored how the WGCN and the final inferred GRNs differ in the application of PBMC and TNBC in terms of both predictive performance and network topologies, as followed:

(1) The difference between WGCN and the final inferred GRNs in inferring performance on six real scRNA-seq datasets. We compared the final GRNs inferred by DeepRIG with the straightforward WGCNA model that simply calculated the Spearman's correlation coefficients. As the weights of WGCN range from 0 to 1, we consider those weights bigger than 0.5 as potential regulations, and reconstruct the GRNs from WGCN. We evaluated two models on six real scRNA-seq datasets. The results demonstrated that the final GRNs inferred by DeepRIG present better performance than reconstructed GRNs from WGCN in terms of AUROC, AUPRC, and EPR values (Fig 6).

(2) We also demonstrated the contrasting network topologies between the WGCN and the final inferred GRNs from PBMCs and TNBC.

Specifically, we visualized the WGCN and the final inferred GRNs for CD14+ Monocytes and CD8 T cells in PBMCs, and analyzed the distributions of various topological attributes (below and Supplementary Fig 6). Our observations revealed distinct characteristics of the final inferred GRNs, including the presence of sub-regulatory networks with central regulators, which were not observed in the WGCN. Furthermore, significant differences in the distributions of several topological attributes such as degree, closeness, hub score, and edge betweenness were observed between the WGCN and the final inferred GRNs. The visualization and analysis of these network structures and topological attributes

suggest that the final inferred GRNs are more effective in identifying hub genes or key regulators. Notably, in CD14+ Monocytes, we identified several regulators, such as CEBPB and STAT1, that exhibited sharp degree changes between the WGCN and the final predicted GRNs. CEBPB was observed to have 101 degrees in the final inferred GRNs but 0 in the WGCN. By comparing its regulatory structures in the GRNs of four cell types of PBMC, we found that CEBPB exhibits a unique regulatory pattern and complex regulatory structure specific to CD14+ monocytes (Fig 7B). This cell-type-specific regulatory structure suggests that CEBPB gene may play a crucial functional role in CD14+ monocytes, as it has been previously demonstrated as a critical TF for monocytic cell proliferation and differentiation[4]. Similarly, STAT1, with sharp degree changes between the WGCN (0 degree) and the final predicted GRN (178 degrees), has been reported as a critical factor driving peak pSTAT1 levels in CD14+ monocytes[5]. In CD8 T cells, we identified JUND as a key regulator, with a degree of 50+ in the final predicted GRN compared to 0 in the WGCN. In fact, JUND is known for its ubiquitous expression during CD8 T cell differentiation[6, 7] and it plays an important role in the response of CD8 T cells to acute infection and cancer[8]. In B cells, we observed a significant degree change between the WGCN and the final inferred GRN for MEF2C, indicating its central role as a transcription factor in the regulatory network. MEF2C has been reported to regulate the calcineurin signaling pathway and is essential for B cell proliferation[9]. The study revealed that MEF2C expression is specific to receptor stimulation in B cells, underscoring its specific regulatory role in receptor-mediated signal transduction.

We further compared the distributions of network topologies between the final predicted GRNs and WGCN of the TNBC scRNA-seq dataset. The results showed significant differences in node degree, closeness, hub score, and edge betweenness (Supplementary Fig. 9). As expected, the distribution of network topologies from the final inferred GRNs exhibited long-tailed characteristics. Ten potential regulators identified in the final inferred GRNs (Fig 8) showed sharp degree changes compared to those in WGCN. In addition, genes including ZNF217, CREBBP, XBP1, RARG, RXRA, E2F4, RAC3, PBX1, and ELF1, also exhibited significant degree changes. Among them, ZNF217[3], CREBBP[10], XBP1[2], RXRA[11], E2F4[12], and PBX1[13] are known to be crucial TFs for breast cancers initiation, progression and metastasis.

Manuscript changes:

-Addition of Supplementary Fig 6:

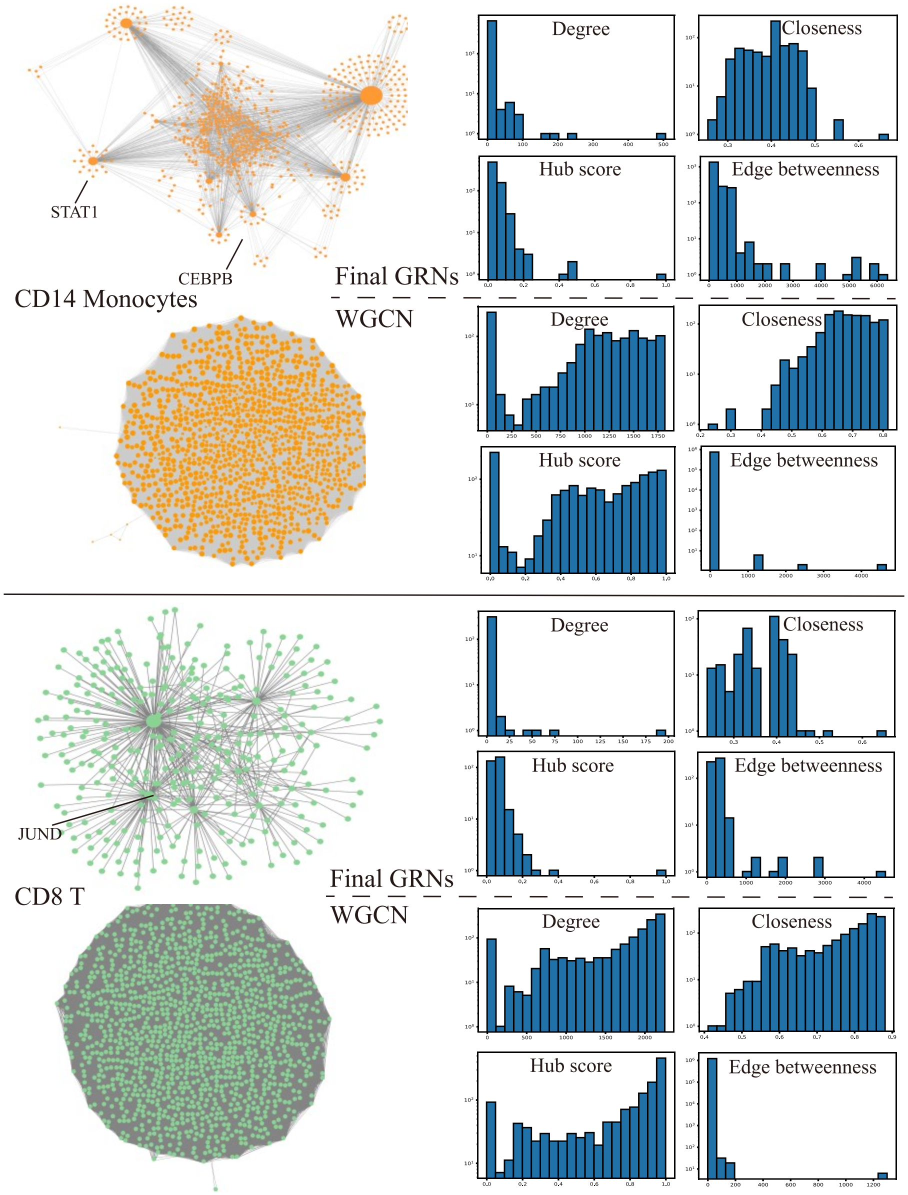

-Addition of following text: "We then explored the differences between the final inferred GRNs and the WGCN from CD14 Monocytes and CD8T cells in PBMCs (Fig S6). As expected, they showed contrasting network topologies in visualizations and the distributions of various topological attributes. Our observations revealed distinct characteristics of the final inferred GRNs, including the presence of sub-regulatory networks with central regulators, which were not observed in the WGCN. Furthermore, significant differences in the distributions of several topological attributes such as degree, closeness, hub score, and edge betweenness were observed between the WGCN and the final inferred GRNs. The visualization and analysis of these network structures and topological attributes suggest that the final inferred GRNs are more effective in identifying hub genes or key regulators."

-Addition of Supplementary Fig 9 (and below):

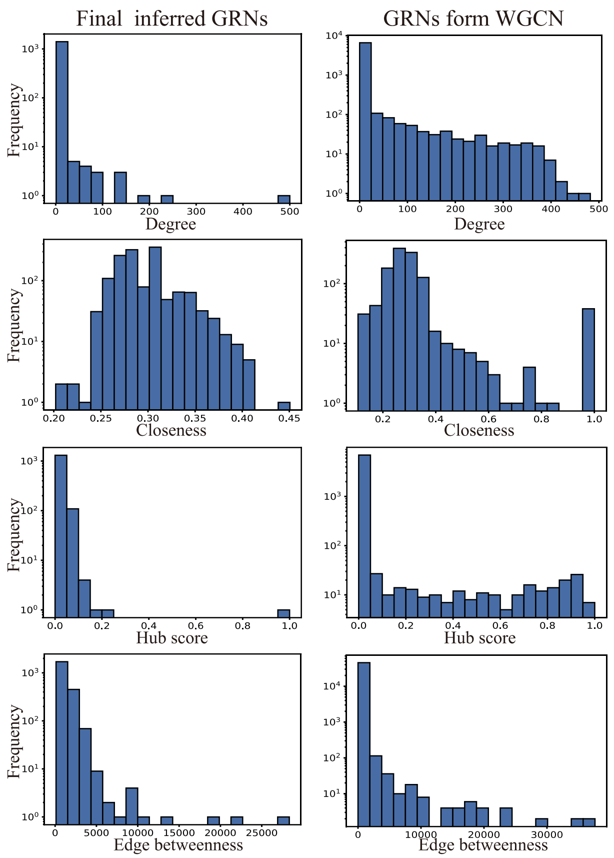

-Addition of following text: "We further compared the distributions of network topologies between the final predicted GRNs and WGCN of the TNBC scRNA-seq dataset. The results showed significant differences in node degree, closeness, hub score, and edge betweenness (Fig S9). As expected, the distribution of network topologies from the final inferred GRNs exhibited long-tailed characteristics. Ten potential regulators identified in the final inferred GRNs (Fig 8) showed sharp degree changes compared to those in WGCN. In addition, genes including ZNF217, CREBBP, XBP1, RARG, RXRA, E2F4, RAC3, PBX1, and ELF1, also exhibited significant degree changes."

2. All the real data comparisons in Figure 5B are unfair because DeepRIG is a supervised model trained on ground truth ChIP-seq data and then evaluated using ChIP-seq data while most of the other methods are unsupervised and do not use ChIP-seq information. This should be clearly stated in the figure and in the main text for this section. Indeed, CNNC, the other supervised method has really similar performance, bringing the question of whether DeepRIG poses a real practical improvement other than being a supervised method.

Authors' response: We thank the reviewer for this important comment. We thank the reviewer for raising this important point. We acknowledge that comparing supervised and unsupervised methods directly may raise concerns about fairness, as they have different access to prior knowledge. It is indeed true that supervised learning methods, such as DeepRIG, CNNC, and 3DCEMA, benefit from the utilization of ground truth ChIP-seq data, which can significantly enhance their performance in GRN inference. We humbly recognize the advantage that prior knowledge provides to supervised methods in terms of performance improvement.

At the same time, it is important to highlight that this advantage does not diminish the value of unsupervised methods. Unsupervised approaches, despite lacking the benefit of ChIP-seq data, still play a crucial role in inferring GRNs and have their own merits in handling diverse biological scenarios where prior knowledge may be limited or unavailable. By presenting the comparison in a balanced and objective manner, we aim to underscore the complementary strengths of both supervised and unsupervised methods in GRN inference.

We have clearly stated and explained it in the figure and in the main text for this section.

Manuscript changes:

Addition of following text: "We acknowledge that comparing supervised and unsupervised methods directly may raise concerns about fairness, as they have different access to prior knowledge. It is indeed true that supervised learning methods, such as DeepRIG, CNNC, and 3DCEMA, benefit from the utilization of ground truth ChIP-seq data, which can significantly enhance their performance in GRN inference. We humbly recognize the advantage that prior knowledge provides to supervised methods in terms of performance improvement. At the same time, it is important to highlight that this advantage does not diminish the value of unsupervised methods. Unsupervised approaches, despite lacking the benefit of ChIP-seq data, still play a crucial role in inferring GRNs and have their own merits in handling diverse biological scenarios where prior knowledge may be limited or unavailable."

Indeed, CNNC, the other supervised method has really similar performance, bringing the question of whether DeepRIG poses a real practical improvement other than being a supervised method.

Authors' response: We thank the reviewer for this comment. While CNNC showed comparable performance in terms of AUROC, our model significantly outperformed it in terms of AUPRC and EPR, which are more suitable metrics for evaluating imbalanced sample classification tasks like GRNs inference. Unlike AUROC, which can lead to misleading evaluations in such scenarios, AUPRC and EPR provide a more accurate assessment of the model's performance on the minority class (i.e. positive samples). Through a meticulous comparison of the three supervised learning methods, DeepRIG exhibited superior performance, proving its practical superiority over CNNC and 3DCEMA, particularly in capturing the regulatory relationships with high precision and recall.

Manuscript changes:

Addition of following text: "While CNNC showed comparable performance in terms of AUROC, our model significantly outperformed it in terms of AUPRC and EPR, which are more suitable metrics for evaluating imbalanced sample classification tasks like GRNs inference. Unlike AUROC, which can lead to misleading evaluations in such scenarios, AUPRC and EPR provide a more accurate assessment of the model's performance on the minority class (i.e. positive samples). Through a meticulous comparison of the three supervised learning methods, DeepRIG exhibited superior performance, proving its practical superiority over CNNC and 3DCEMA, particularly in capturing the regulatory relationships with high precision and recall."

3. Although the authors explain in lines 565-569 that they only considered undirected regulatory networks because the ChIP-seq data they use does not specify activation or inhibition, single-cell gene expression networks do indirectly contain that information. No directionality nor inhibitory relationships are considered in the current model. In fact, many of the nodes in the presented networks from Figure 3 have

been shown to have mutually inhibitory relationships, such as Gata1 and Pu1 in the HSC network. The authors could improve the potential of their manuscript by commenting/expanding their conclusions to positive and negative regulation, which is widespread in real biological networks.

Authors' response: We appreciate the valuable suggestion from the reviewer. Among nine baselines, several GRNs inference methods, including PIDC, GRNBOOST2, and DeepSEM, output unsigned regulatory networks, making it challenging to differentiate between activating and inhibitory regulations. Moreover, the ground truth ChIP-seq data also provides unsigned regulatory associations, further complicating the evaluation. To address this limitation, we focused on evaluating unsigned GRNs of three types of ground truth for all baseline methods.

In contrast, our model provides a significant advantage by outputting signed regulatory networks, representing both activating and inhibitory regulations, which closely mirror the complexity of real biological networks. A positive score in the predicted interaction indicates an activating regulation, while a negative score signifies an inhibitory one. To thoroughly assess the performance of DeepRIG, PPCOR, LEAP, SCODE, SCRIBE, and CNNC in predicting activating and inhibitory regulations, we conducted an in-depth investigation on four curated networks (Supplementary Fig 2). We designed an approach where activating regulations were assigned label 1, inhibitory regulations were labeled -1, while 0 denotes no regulation. We then calculated the root mean square error (RMSE) between the predicted signed GRNs and the corresponding ground truth labels. The results indicated that DeepRIG achieved the smallest RMSE on HSC, VSC, and GSD networks, and the second-smallest RMSE on the mCAD network. These findings suggest that our model outperformed the other baseline methods in predicting both activating and inhibitory regulations.

Manuscript changes:

-Addition of Supplementary Fig 2 and below:

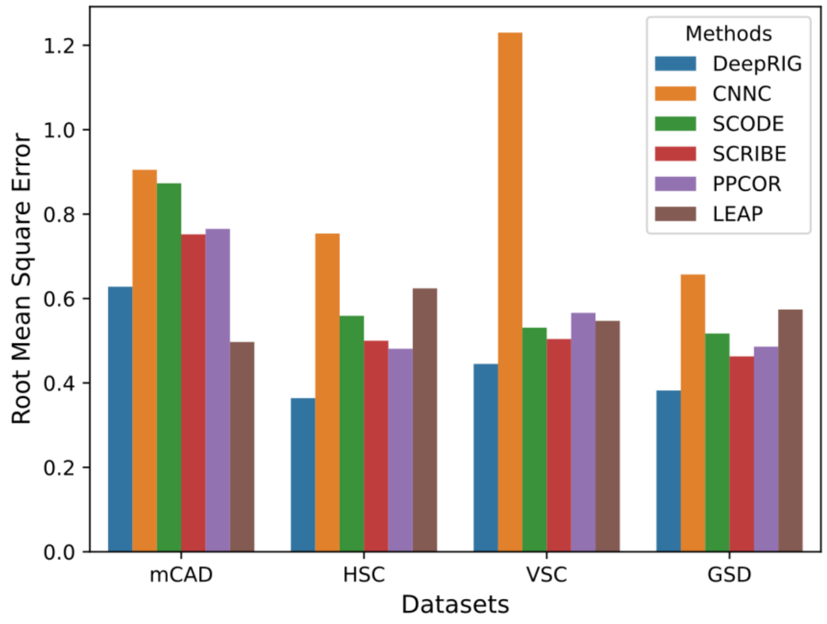

-Addition of following text: “Several GRNs inference methods, including PIDC, GRNBOOST2, and DeepSEM, output unsigned regulatory networks, making it challenging to differentiate between activating and inhibitory regulations. Moreover, the ground truth ChIP-seq data also provides unsigned regulatory associations, further complicating the evaluation. To address this limitation, we focused on evaluating unsigned GRNs of three types of ground truth for all baseline methods.

In contrast, our model provides a significant advantage by outputting signed regulatory networks, representing both activating and inhibitory regulations, which closely mirror the complexity of real biological networks. A positive score in the predicted interaction indicates an activating regulation, while a negative score signifies an inhibitory one. To thoroughly assess the performance of DeepRIG, PPCOR, LEAP, SCODE, SCRIBE, and CNNC in predicting activating and inhibitory regulations, we conducted an in-depth investigation on four curated networks (Fig S2). We designed an approach where activating regulations were assigned label 1, inhibitory regulations were labeled -1, while 0 denotes no regulation. We then calculated the root mean square error (RMSE) between the predicted signed GRNs and the corresponding ground truth labels. The results indicated that DeepRIG achieved the smallest RMSE on HSC, VSC, and GSD networks, and the second-smallest RMSE on the mCAD network. These findings suggest that our model outperformed the other baseline methods in predicting both activating and inhibitory regulations.”

4. Line 154: “to better mimic the zero-flame”. Are the authors referring to “Zero-inflation”? It would be most relevant to model the number of UMIs detected per single-cell too. The number of UMIs (or number of genes, which is positively correlated with the former) is, together with the number of single-cells, an important driver of the sparsity in single-cell datasets.

Authors’ response: We thank the reviewer for bringing the spelling mistake to our attention. We apologize for the error and appreciate the reviewer's clarification regarding the term "Zero-inflation". As mentioned by the reviewer, sparsity is a prominent feature of single-cell datasets. Due to the inherent heterogeneity and sparsity among cells, many genes may not be detected in certain cells or may have very few unique molecular identifiers (UMIs) detected. This leads to the presence of zero values in the single-cell data, indicating the zero-inflation characteristic of the dataset. Therefore, in order to accurately model the number of UMIs detected per cell, it is essential to account for the zero-inflation characteristics observed in single-cell data.

5. Figure 4. Please change the legend from “no. of cells” to “Dropout rate”.

Authors’ response: We appreciate the reviewer for bringing to our attention the mistake in Fig 4. We sincerely apologize for the oversight and have promptly made the necessary correction, revising the legend to "Dropout rate".

Manuscript changes:

Modification of Fig 4.

6. The numbers of cells in figure 5C is very low for current datasets. The authors should benchmark or show how their method scales up this with tens of thousands or hundreds of thousands of cells, which are now routine in single-cell experiments. Similarly, the number of genes detected in an experiment can reach 8000 or more.

Authors' response: We thank the reviewer for raising this important point. We applied our model to the whole PBMC8k dataset with 8339 cells to demonstrate its scalability in handling larger cell numbers. While we acknowledge that the PBMC8k dataset is not in the tens of thousands or millions, this analysis allowed us to evaluate the time complexity and scalability of our approach and other baseline methods on relatively larger datasets. Additionally, generating a suitable ground truth for data sets of tens of thousands or millions of cells is somewhat challenging. We also compared our model to PIDC, PPCOR, GRNBOOST2, DeepSEM, CNNC which do not require peseudotime or stamped information. As expected, PPCOR only took 1 minute to predict GRNs, and DeepRIG and DeepSEM completed the GRN inference in just 4 minutes and 10 minutes, respectively. On the other hand, PIDC and CNNC required 60 to over 100 minutes to generate the outputs, while GRNBOOST2 took thousands of minutes. Regarding gene scalability, we evaluated our model and five baselines on the TNBC scRNA-seq dataset containing 7185 genes. Notably, PPCOR completed predictions in 22 minutes, while DeepRIG and DeepSEM took 61 and 140 minutes, respectively, for GRN inference. GRNBOOST2 required 1383 minutes, and PIDC failed to provide predictions. This comparison highlights the scalability of our model in handling datasets with a large number of cells and genes. Additionally, we observed that the time complexity of most methods is primarily influenced by the number of genes.

Manuscript changes:

-Addition of following text: "We further applied our model to the whole PBMC8k dataset with 8339 cells to demonstrate its scalability in handling larger cell numbers. We also compared our model to PIDC, PPCOR, GRNBOOST2, DeepSEM, CNNC which do not require peseudotime or stamped information. As expected, PPCOR only took 1 minute to predict GRNs, and DeepRIG and DeepSEM completed the GRN inference in just 4 minutes and 10 minutes, respectively. On the other hand, PIDC and CNNC required 60 to over 100 minutes to generate the outputs, while GRNBOOST2 took thousands of minutes. This comparison clearly demonstrated the scalability of our model when dealing with datasets containing a large number of cells. Regarding gene scalability, we evaluated our model and five baselines on the TNBC scRNA-seq dataset containing 7185 genes. Notably, PPCOR completed predictions in 22 minutes, while DeepRIG and DeepSEM took 61 and 140 minutes, respectively, for GRN inference. GRNBOOST2 required 1383 minutes, and PIDC failed to provide predictions. This comparison highlights the scalability of our model in handling datasets with a large number of cells and genes. Additionally, we observed that the time complexity of most methods is primarily influenced by the number of genes."

7. The authors should also include the results from the other network inference methods in figure 7, and comment on how they differ.

Authors' response: We thank the reviewer for raising this important point. We compared the performance of several other GRN inference methods, including PIDC, PPCOR, GRNBOOST2, DeepSEM, and CNNC, with our proposed DeepRIG method on CD14 Monocytes of PBMCs. Visualizing and analyzing the network structures of the inferred GRNs from these methods, as well as DeepRIG, revealed interesting findings (below and Supplementary Fig. 7). We observed that the GRNs inferred by CNNC and GRNBOOST2 exhibited similarities to the predictions of our model, albeit with a more dispersed network structure. On the other hand, the GRNs predicted by the correlation-based methods, PIDC and PPCOR, were found to be more akin to the WGCN. Notably, significant differences were observed among these methods in terms of topological attributes, such as degrees, closeness, hub scores, and edge betweenness. A particularly intriguing observation was that the inferred GRNs from our model and CNNC exhibited a power-law

distribution, resembling scale-free networks. In scale-free networks, a few key hub nodes have rich connections to other nodes, while most nodes have limited connections. This distribution pattern is commonly observed in various real biological networks, including metabolic networks[14, 15], protein-protein interaction networks[16], and transcriptional regulation networks[17]. Our analysis suggests that the GRNs inferred by our model better capture the regulatory relationships present in the real data and are more effective in identifying hub genes or regulators.

Manuscript changes:

-Addition of supplementary Fig 7:

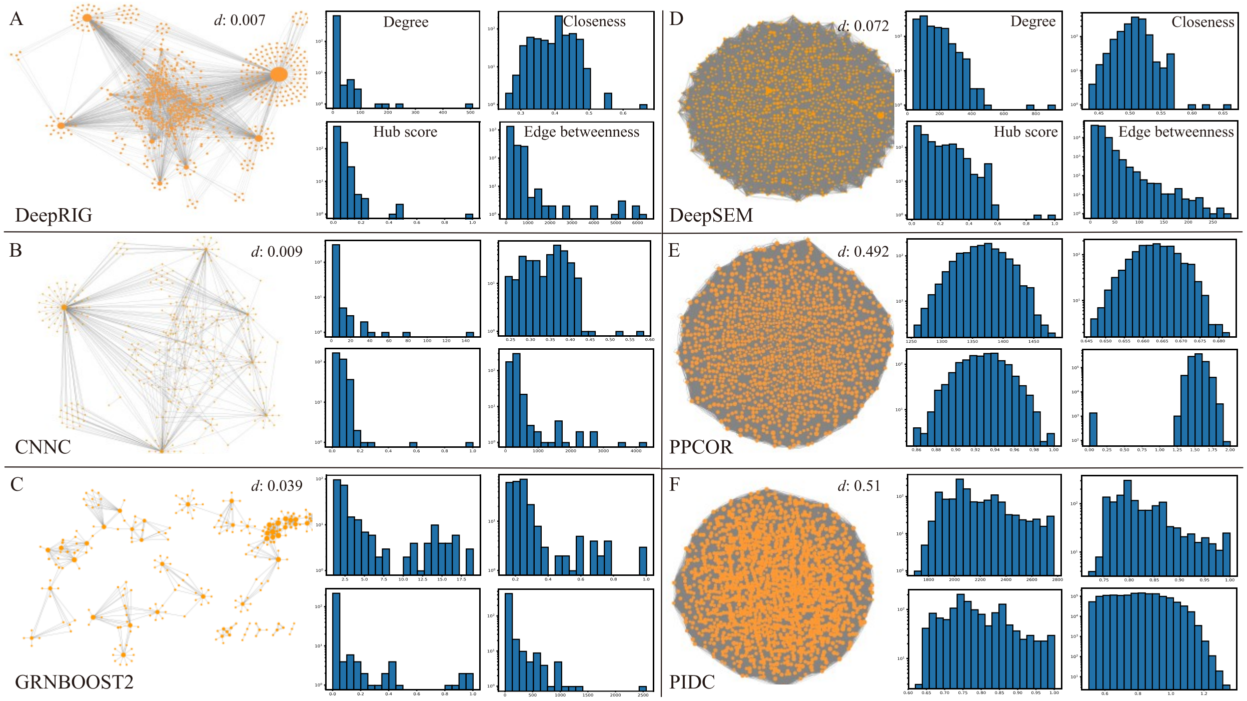

-Addition of the following text: "We compared the performance of several other GRN inference methods, including PIDC, PPCOR, GRNBOOST2, DeepSEM, and CNNC, with our proposed DeepRIG method on CD14 Monocytes of PBMCs. Visualizing and analyzing the network structures of the inferred GRNs from these methods, as well as DeepRIG, revealed interesting findings (Fig. S7). We observed that the GRNs inferred by CNNC and GRNBOOST2 exhibited similarities to the predictions of our model, albeit with a more dispersed network structure. On the other hand, the GRNs predicted by the correlation-based methods, PIDC and PPCOR, were found to be more akin to the WGCN. Notably, significant differences were observed among these methods in terms of topological attributes, such as degrees, closeness, hub scores, and edge betweenness. A particularly intriguing observation was that the inferred GRNs from our model and CNNC exhibited a power-law distribution, resembling scale-free networks. In scale-free networks, a few key hub nodes have rich connections to other nodes, while most nodes have limited connections. This distribution pattern is commonly observed in various real biological networks, including metabolic networks, protein-protein interaction networks, and transcriptional regulation networks. Our analysis suggests that the GRNs inferred by our model better capture the regulatory relationships present in the real data and are more effective in identifying hub genes or regulators."

8. Why are only two TFs discussed in figure 7? How about the other TFs expressed in this dataset?

Authors' response: We appreciate the reviewer's valuable comment. Our approach focuses on uncovering the distinct gene regulation mechanisms exhibited by cells with varying transcription levels. In Figure 7, our goal was to provide cell-type-specific GRN inference and identify regulators that are specific to each cell type. In the manuscript, we highlighted two regulators, CEBPB and SPI1, which were found to exhibit cell-type-specific regulatory structures in CD14 Monocytes, as well as two regulators, MEF2C and EGR1, in B cells.

We acknowledge the reviewer's suggestion that additional discussion is needed to cover more cell-type-specific regulators identified by our model, as they present significant differences in regulatory structures across cell types. Therefore, we have included introductions of several additional cell-type-specific regulators identified by our model, such as GATA2 [Hsu et al., Blood 118(10): 2653, (2011)], STAT1 [Zimmerman et al., Front Immunol 10, (2019)] for CD14 Monocytes, PAX5 [Cobaleda et al., Nat Immunol 8: 463, (2007)] and RUNX3 [Spender et al., Oncogene 24, 1873 (2005)] for B cells. We have also provided a comprehensive list of all identified cell-type-specific regulators in the corresponding predicted GRNs (Supplementary Table 7).

We would like to express our gratitude for the reviewer's insightful suggestion, which has enhanced the clarity and completeness of our manuscript.

Manuscript changes:

-Addition of the following text: "We have also identified GATA2 and STAT1 as two specific regulators that exhibit cell-type-specific regulatory structures in CD14 Monocytes. GATA2 demonstrated 201 degrees in CD14 Monocytes, while only 3 degrees in B cells. Furthermore, GATA2 exhibited the highest Betweenness Centrality value (0.59), indicating its significant influence and control over other genes. GATA2 is recognized as an important TF involved in regulating various biological processes, including ontogeny in monocytes. Similarly, STAT1, which showed sharp degree changes across cell types, has been reported as a critical factor driving peak pSTAT1 levels in CD14+ monocytes. In the inferred GRNs of B cells, two TFs, PAX5 and RUNX3 were also identified as a cell-type-specific regulator by our model. Indeed, the well-established transcription factor PAX5 plays a pivotal role in governing the identity and function of B cells throughout B lymphopoiesis and has been associated with human B cell malignancies. RUNX3 is known for its cross-regulation mechanism with RUNX1 in B cells, and its expression is required for efficient B cell proliferation. The comprehensive list of identified regulators specific to each cell type can be found in Table S7."

-Addition of Supplementary Table 7.

Cell type	Identified regulators
CD14 Monocytes	GATA2, ATF3, MYB, JUND, PAX5, BACH2, BATF, GATA3, MYC, STAT1, CEBPB, SPI1, RPS4Y2, FCER1A, BCL11A
B cells	EGR1, EGR2, TBX21, MEF2C, GATA2, BACH2, STAT1, MYC, NFE2, PAX5, RUNX3, FCER1A
CD4T cells	GPR82, BATF, TRBC1, MYB, SPI1, EGR1, LINC01550, SCGB3A1
CD8T cells	BACH2, NFE2, SPI1, MAX, GATA2, MEF2C, GATA3, ATF3

Minor comments

-line 297: what do the authors mean by "top-four cell numbers"?

Authors' response: We thank the reviewer for pointing out this inappropriate expression. We actually mean that four cell clusters with highest cell numbers. We have corrected this inappropriate expression in the article.

Manuscript changes:

Addition of the following text: "As a result, we focused on four cell clusters with the highest cell numbers, namely CD4+ T cells, CD14+ Monocyte cells, CD8+ T cells, and B cells, for cell-type-specific GRN inference."

-Please check inconsistent grammar throughout the whole manuscript. A couple of examples: "PPCOR has the best computational complexity and only spent only a few seconds to complete the GRN ", or "Due to the sizes of scRNA-seq datasets vary from hundreds to millions, ".

Authors' response: We thank the reviewer for this comment. We have checked inconsistent grammar throughout the whole manuscript and corrected them.

References:

1. Nechanitzky R, Akbas D, Scherer S, Györy I, Hoyler T, Ramamoorthy S, et al. Transcription factor EBF1 is essential for the maintenance of B cell identity and prevention of alternative fates in committed cells. Nat Immunol. 2013;14(8):867-875.
2. Chen X, Iliopoulos D, Zhang Q, Tang Q, Greenblatt MB, Hatziapostolou M, et al. XBP1 promotes triple-negative breast cancer by controlling the HIF1α pathway. Nature. 2014;508(7494):103-107.
3. Zhang C, Zhi WI, Lu H, Samanta D, Chen I, Gabrielson E, et al. Hypoxia-inducible factors regulate pluripotency factor expression by ZNF217- and ALKBH5-mediated modulation of RNA methylation in breast cancer cells. Oncotarget. 2016;7(40):64527-64542.
4. Huber R, Pietsch D, Panterodt T, Brand K. Regulation of C/EBPβ and resulting functions in cells of the monocytic lineage. Cell Signal. 2012;24(6):1287-1296.
5. Zimmerman O, Olbrich P, Freeman AF, Rosen LB, Uzel G, Zerbe CS, et al. STAT1 Gain-of-Function Mutations Cause High Total STAT1 Levels With Normal Dephosphorylation. Front Immunol. 2019;10.
6. Scharer CD, Barwick BG, Youngblood BA, Ahmed R, Boss JM. Global DNA Methylation Remodeling Accompanies CD8 T Cell Effector Function. The Journal of Immunology. 2013;191(6):3419-3429.
7. Ruppert SM, Chehtane M, Zhang G, Hu H, Li X, Khaled AR. JunD/AP-1-Mediated Gene Expression Promotes Lymphocyte Growth Dependent on Interleukin-7 Signal Transduction. PLoS One. 2012;7(2):e32262.
8. Papavassiliou AG, Musti AM. The Multifaceted Output of c-Jun Biological Activity: Focus at the Junction of CD8 T Cell Activation and Exhaustion. Cells [Internet]. 2020; 9(11).
9. Wilker PR, Kohyama M, Sandau MM, Albring JC, Nakagawa O, Schwarz JJ, et al. Transcription factor Mef2c is required for B cell proliferation and survival after antigen receptor stimulation. Nat Immunol. 2008;9(6):603-612.

10. Peck B, Bland P, Mavrommati I, Muirhead G, Cottom H, Wai PT, et al. 3D Functional Genomics Screens Identify CREBBP as a Targetable Driver in Aggressive Triple-Negative Breast Cancer. Cancer Res. 2021;81(4):847-859.
11. Gorbacheva AM, Uvarova AN, Ustiugova AS, Bhattacharyya A, Korneev KV, Kuprash DV, et al. EGR1 and RXRA transcription factors link TGF-β pathway and CCL2 expression in triple negative breast cancer cells. Sci Rep. 2021;11(1):14120.
12. Macaluso M, Cinti C, Russo G, Russo A, Giordano A. pRb2/p130-E2F4/5-HDAC1-SUV39H1-p300 and pRb2/p130-E2F4/5-HDAC1-SUV39H1-DNMT1 multimolecular complexes mediate the transcription of estrogen receptor-α in breast cancer. Oncogene. 2003;22(23):3511-3517.
13. Magnani L, Ballantyne EB, Zhang X, Lupien M. PBX1 Genomic Pioneer Function Drives ERα Signaling Underlying Progression in Breast Cancer. PLoS Genet. 2011;7(11):e1002368.
14. Jeong H, Tombor B, Albert R, Oltvai ZN, Barabási AL. The large-scale organization of metabolic networks. Nature. 2000;407(6804):651-654.
15. Almaas E, Barabási A-L. Power Laws in Biological Networks. In: Koonin EV, Wolf YI, Karev GP, editors. Power Laws, Scale-Free Networks and Genome Biology. Boston, MA: Springer US; 2006. p. 1-11.
16. Ueda HR, Hayashi S, Matsuyama S, Yomo T, Hashimoto S, Kay SA, et al. Universality and flexibility in gene expression from bacteria to human. Proceedings of the National Academy of Sciences. 2004;101(11):3765-3769.
17. Ouma WZ, Pogacar K, Grotewold E. Topological and statistical analyses of gene regulatory networks reveal unifying yet quantitatively different emergent properties. PLoS Comput Biol. 2018;14(4):e1006098.
