# Supplementary material for: Inferring gene regulatory network from single-cell transcriptomes with graph autoencoder model
Source: PLoS Genet. 2023 Sep 13;19(9):e1010942. doi: 10.1371/journal.pgen.1010942 (PMC10519590; doi:10.1371/journal.pgen.1010942)
Supplement: Attachment — Submitted filename: reviewer comments.docx [file pgen.1010942.s018.docx]

Jiacheng Wang et al. employed a graph autoencoder-based method to infer gene regulatory networks from single-cell RNA sequencing data. The model aims to elucidate the global regulatory structure rather than merely the regulatory relationships between gene pairs. I believe this study is one step further on GRN reconstruction and for genomic research. However, the following concerns need to be resolved to underscore the value of this study:

1. In the introductory section, since the authors have already mentioned tools that utilize RNA velocity and ODE models to infer gene regulatory networks, it would also be relevant to discuss very briefly the following works that employ similar data/models that could to some extent infer GRNs:

Qiu, X., et al. (2022). "Mapping transcriptomic vector fields of single cells." Cell.

Schiebinger, G., et al. (2019). "Optimal-Transport Analysis of Single-Cell Gene Expression Identifies Developmental Trajectories in Reprogramming." Cell.

2. The impact of the presented research appears constrained due to the reliance on pre-existing “gold-standard” networks for training data. Although I understand that the model only needs to be fed with a fraction of known interactions, I think it would enhance the value of this tool if the authors could demonstrate that a trained model can be transferred to other cell types, conditions, or even datasets. The transferability of the method is somewhat demonstrated using the breast cancer dataset, where the model is trained with GRN of normal breast cells. This point is, however, not highlighted or further explored enough in my opinion. For instance, it would be compelling to see if the model trained on one or multiple cell types of the PBMC dataset could be applied to a different, untrained cell type.

3. In the TNBC dataset it seems to me that 14,915 interactions are used to train the model, while it predicted 173,399 interactions, which is 10+ times higher. Are the trained interactions all included in the predicted interactions? How confident are the newly predicted interactions? Is there any example of predicted interactions that are not in the training data validated through other experiments?

4. When using simulated and real data for the performance comparison, my understanding is that the underlying networks and Chip-seq validated interactions are used to train deepRIG. This makes the comparison not completely fair since other methods may not be trained on network structures but only on expression data (e.g. deepSEM). This should be addressed or clearly explained in the result section. In addition, the authors may consider using the simulated data to demonstrate what portion of all the interactions should be used to train the model in order for it to make accurate predictions.

5. In figure 8B and related paragraphs, the authors should briefly explain the meanings of betweenness and pagerank scores and what biological properties do they describe about the genes.
